# A climate-driven mechanistic population model of *Aedes albopictus* with diapause

**DOI:** 10.1186/s13071-016-1448-y

**Published:** 2016-03-24

**Authors:** Pengfei Jia, Liang Lu, Xiang Chen, Jin Chen, Li Guo, Xiao Yu, Qiyong Liu

**Affiliations:** College of Global Change and Earth System Science, Beijing Normal University, Beijing, 100875 China; State Key Laboratory of Infectious Disease Prevention and Control, Collaborative Innovation Center for Diagnosis and Treatment of Infectious Diseases, National Institute for Communicable Disease Control and Prevention, China CDC, Beijing, 102206 China; State Key Laboratory of Earth Surface Processes and Resource Ecology, Beijing Normal University, Beijing, 100875 China; Department of Emergency Management, Arkansas Tech University, Russellville, Arkansas 72801 USA

**Keywords:** *Aedes albopictus*, Population dynamics, Mechanistic model, Climate-driven, Diapause

## Abstract

**Background:**

The mosquito *Aedes albopitus* is a competent vector for the transmission of many blood-borne pathogens. An important factor that affects the mosquitoes’ development and spreading is climate, such as temperature, precipitation and photoperiod. Existing climate-driven mechanistic models overlook the seasonal pattern of diapause, referred to as the survival strategy of mosquito eggs being dormant and unable to hatch under extreme weather. With respect to diapause, several issues remain unaddressed, including identifying the time when diapause eggs are laid and hatched under different climatic conditions, demarcating the thresholds of diapause and non-diapause periods, and considering the mortality rate of diapause eggs.

**Methods:**

Here we propose a generic climate-driven mechanistic population model of *Ae. albopitus* applicable to most *Ae. albopictus*-colonized areas. The new model is an improvement over the previous work by incorporating the diapause behaviors with many modifications to the stage-specific mechanism of the mosquitoes’ life-cycle. monthly Container Index (CI) of *Ae. albopitus* collected in two Chinese cities, Guangzhou and Shanghai is used for model validation.

**Results:**

The simulation results by the proposed model is validated with entomological field data by the Pearson correlation coefficient *r*^2^ in Guangzhou (*r*^2^ = 0.84) and in Shanghai (*r*^2^ = 0.90). In addition, by consolidating the effect of diapause-related adjustments and temperature-related parameters in the model, the improvement is significant over the basic model.

**Conclusions:**

The model highlights the importance of considering diapause in simulating *Ae. albopitus* population. It also corroborates that temperature and photoperiod are significant in affecting the population dynamics of the mosquito. By refining the relationship between *Ae. albopitus* population and climatic factors, the model serves to establish a mechanistic relation to the growth and decline of the species*.* Understanding this relationship in a better way will benefit studying the transmission and the spatiotemporal distribution of mosquito-borne epidemics and eventually facilitating the early warning and control of the diseases.

## Background

*Aedes albopitus* (Skuse), also known as the Asian tiger mosquito, is a competent vector for the transmission of many blood-borne epidemics such as dengue fever, West Nile virus infections and Chikungunya fever [1–5]. *Ae. albopictus* is a species native to the tropical areas of Southeast Asia; and during the last century, it has rapidly invaded countries throughout the world. Now the species is pervasively found in the subtropical and temperate climate areas of East Asia, Europe, Africa, the Middle East and the Americas [1, 2]. The extensive spreading of *Ae. albopictus* is not only prompted by the increasing trend of international trade and travel [3–5] but is also mediated by climate change in a global context [6]. It has been corroborated that the growth of *Ae. albopictus* is constrained by the changing nature of physical environment; as a result, their population density is extrinsically impinged by a series of climatic factors including temperature, precipitation and photoperiod [7]. Because of this link to climate change, modeling the population dynamics of *Ae. albopictus* based on climatic factors serves a critical role for further identifying the causal relation to the transmission and control of mosquito-borne pathogens [8].

Within the realm of quantitative modeling, the climate-driven dynamics of the mosquito has been explored by two types of models: the statistical population model and the mechanistic population model [9]. The statistical population model aims to establish a mathematical correlation between the population abundance and climatic factors using data solicited from controlled experiments or field observations [10–12]. Although these statistical relationships are relatively straightforward and can be easily understood, they are flawed in describing the intrinsic biological mechanism of how the morphology of pathogen carriers is mediated by the environment. Another overlooked facet in the statistical population model pertains to the limited coverage of the species’ life-cycle stages; for example, the majority of mosquito models have only differentiated between the aquatic period and the aerial period, while other sub-stages (e.g. eggs, larvae, pupae) have been less emphasized [13]. When climatic factors are involved in promoting or inhibiting the development, the influence on each sub-stage of the life-cycle is a critical aspect that must be further scrutinized [14, 15]. To overcome these limitations, the mechanistic population model explores the fluctuation of population by factoring in *a priori* established development process in a context constrained by environment [8]. For the study of *Ae. albopictus*, this type of model and its variations have been applied to specific geographic regions and have been modified for extensions to other *Aedes* spp. [16–22]. One important work in this area is attributed to Erickson et al. [23], who developed a stage-structured population model consisting of six ordinary differential equations to correspond to different stages of *Ae. albopictus’* life-cycle, with each equation measuring the mortality rate and growth rate dependent on a stage-specific temperature variable. Thereafter, Cailly et al. [24] established a generic temperature-driven model that extended the application to different mosquito species. This model served a more general purpose to represent the complete life-cycle with ten model compartments and temperature-driven mortality rate and growth rate [25, 26]. In a follow-up study, Tran et al. [27] optimized the parameters and transition functions in Cailly’s model to estimate the population dynamics of *Ae. albopictus* with improved accuracy and a better fit to field observations.

An overlooked area in the formulation of the mechanistic population model is the phenomenon of diapause. Diapause refers to the physiological mechanism within certain species that inhibits the development of organism as a strategy to survive unfavorable environmental conditions, such as extreme weather [28, 29]. Diapause is observed among *Ae. albopictus* in selected subtropical areas and temperate climate areas where the relatively cold season of a year makes the mosquito eggs become dormant and unable to hatch [7, 30]. The delay in development can only be remedied when the eggs are exposed to enough warmth or a long photoperiod [1, 7]. Many entomologists previously associated *Ae. albopictus* diapause with environmental conditions using controlled experiments. For example, Wang [31] and Imai & Maeda [32] discovered that the intervening factors of diapause included low temperatures and short photoperiods; and this conclusion was later confirmed by a series of controlled experiments [1, 7, 30, 33]. In addition, comparative studies identified that the unique mechanism of diapause greatly improved the survival rate of *Ae. albopictus* under extreme desiccation and cold-stress conditions [34, 35]. These findings, however, have been rarely incorporated in the mechanistic population model [27]. Two exceptions are the models proposed by Cailly et al. [24] and Tran et al. [27], where diapause was quantified as a piecewise function dichotomizing the development into diapause period, say last September through early March, and that of non-diapause for the rest of year. Compared with Erickson et al. [23], accounting for diapause greatly improved the model performance and generated a close approximation of the field observation. However, the adjustment was based on a relative empirical assumption that diapause occurs in the same time period over different years as well as does not manifest regional differences. As diapause is a dynamic process primarily dictated by degrees of temperature and lengths of photoperiod [31, 32], it is more reasonable to assume diapause as a temperature- and photoperiod-driven phenomenon in a general way to consolidate the effect of temporal as well as regional differences.

Along the line of existing mechanistic population models [23, 24, 27], this paper, proposes a generic model to study *Ae. albopictus* population by considering diapause as a dynamic process conditional on the change of temperature and photoperiod. By restructuring the model and tuning the parameters, the study strictly captures the stage-specific mechanism of the mosquitoes’ life-cycle. In addition, the model has been validated by field data collected in two Chinese cities over a five-year span where the phenomenon of diapause was introduced by seasonality. The proposed model aims to: (i) define a fine-tuned relationship between major climatic variables (temperature and photoperiod) and diapause-related events in *Ae. albopictus’*s development cycle; (ii) explicitly quantify the effect of temperature on the population dynamics during the aquatic period based on controlled laboratory experiments and related literature.

The paper is organized as follows. The Methods section first presents the two sets of field observations in Chinese cities, which are employed as evidence for model validation. Then the new model is proposed based on two existing mechanistic population models. The Results section demonstrates simulation results, which are further compared and validated with field observations. The Discussion section discusses the improvement of the model over the original model as well as the sensitivity of non-climatic variables in the model. Lastly, the Conclusions section summarizes the contribution of the study and proposes directions for future research.

## Methods

### Study areas and data

To validate the model, we firstly conducted rigorous field experiments to collect *Ae. albopictus* larval samples. Field observations on the *Ae. albopictus* population were conducted in two southern Chinese cities, Guangzhou and Shanghai, over a respective period of five years. The two study areas both belong to the subtropical climate zone with a humid and hot summer and an arid and cold winter. Compared to Guangzhou, the weather in Shanghai exhibits a higher degree of seasonality with a colder spring, autumn and winter.

The field observations were quantified by the monthly Container Index (CI) of *Ae. albopictus* larvae in Guangzhou (2007–2011) and Shanghai (2009–2013) collected by the China Centre for Disease Control and Prevention (CDC), as shown in Table 1. To acquire the data, we distributed 50–100 sampling containers with clean water in outdoor conditions and then checked the larval density at the end of each sampling period. Generally, the sampling was conducted every 3–5 days in areas where cases of dengue fever were observed and every 15 days in other areas. The corresponding CI was calculated by Equation 1, where *N*^*+*^denotes number of containers with at least one *Ae. albopictus* larva and *N* denotes the number of total sampling containers [36]. Then we consolidated the CI values collected in different sampling areas into a monthly index. This monthly CI represents the monthly *Ae. albopictus* population abundance at the larval stage and serves as the field evidence for model validation.

**Table 1:** Monthly CI (in %) of *Ae. albopictus* larvae in Guangzhou and in Shanghai over a respective five-year period

	Guangzhou					Shanghai				
Month	2007	2008	2009	2010	2011	2009	2010	2011	2012	2013
1				0.27						
2										
3	0.32		5.73	1.08	0.46					
4	3.35	2.50	6.23	2.10	6.42	0.45	0.90	0.67	0.84	0.93
5	7.06	9.58	9.41	16.68	8.73	2.87	2.41	1.93	2.05	3.20
6	16.11	13.06	12.61	11.18	9.55	5.05	3.87	6.41	5.69	6.87
7	14.11	13.01	16.23	10.48	8.14	5.31	3.39	7.01	6.69	5.88
8	6.70	15.02	11.28	12.03	10.31	6.58	4.50	6.71	5.74	5.54
9	8.40	15.41	11.20	10.93	11.21	5.16	3.97	5.00	5.42	4.17
10	4.53	18.52	7.37	4.80	8.96	2.80	2.01	2.22	2.17	2.56
11	2.17	3.91	0.59	2.34	6.35	0.29	1.05	1.29	0.62	0.86
12		1.85	0.53	1.16	4.79					

Note: A blank slot indicates the observed larvae during the month is close to 0 (CI ≈ 0.00%)

1 $$ CI=\frac{N^{+}}{N}\times 100\%. $$

To correspond to the CI data for each city, we collected daily mean temperature and precipitation data from China Meteorological Data Sharing Service System. We also derived photoperiod data using an existing source [37]. These datasets included Guangzhou over 2007–2011 and Shanghai over 2009–2013.

### Basic mechanistic population model

**Fig. 1:**
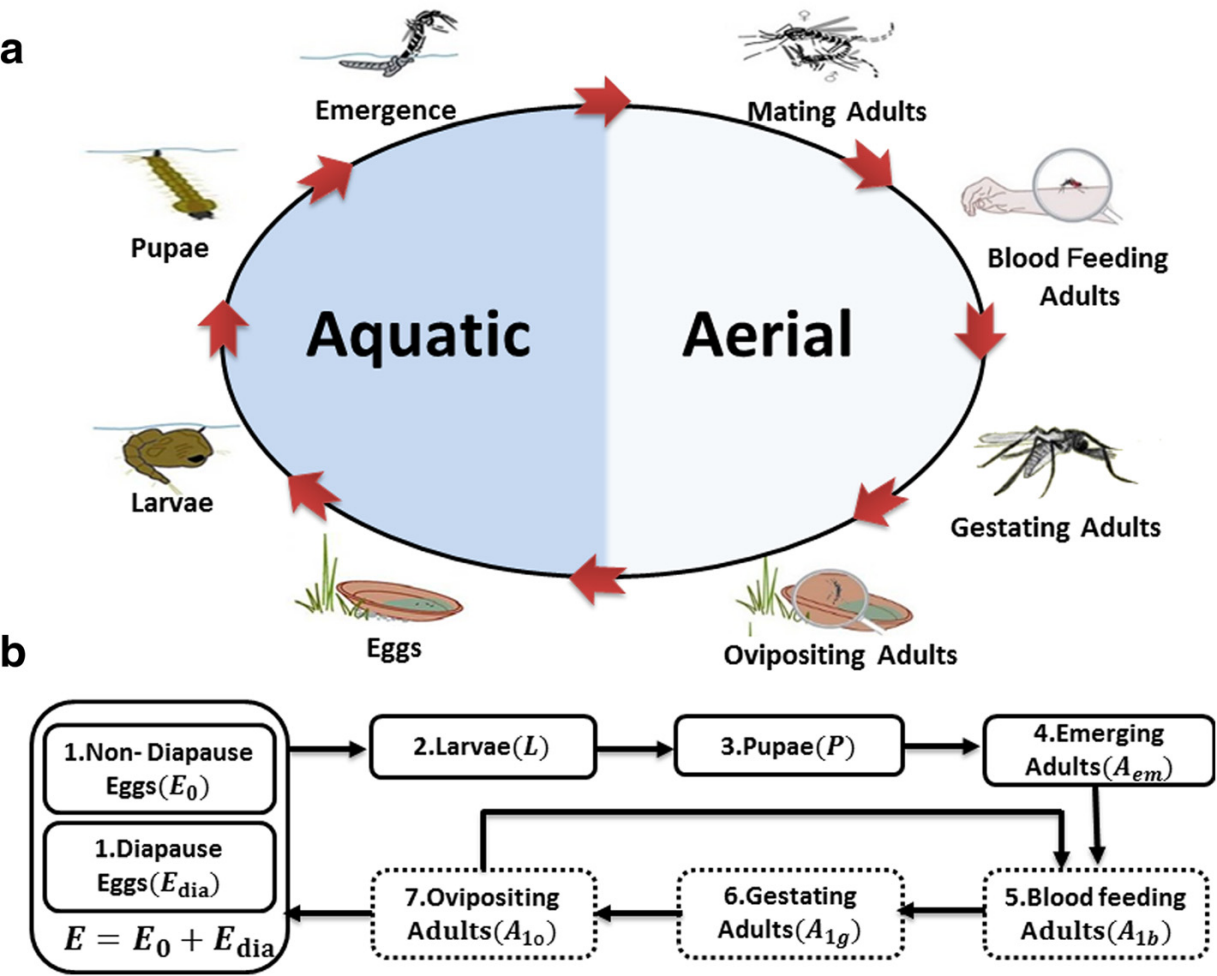
**a** The eight stages of *Ae. albopcitus* life-cycle. **b** The basic seven-stage mechanistic population model, where the emergence and mating adults are combined into the emerging adult stage. Dotted boxes indicate three adult stages

Our proposed model is a natural extension of two basic mechanistic population models proposed by Cailly *et al.* [24] and Tran et al. [27]. These two models were formulated by considering the life-cycle of *Ae. albopictus* that consists of two principal periods by distinct habitats: the aquatic period and the aerial period, as shown in Fig. 1a. The two periods can be further divided into the following eight sub-stages: four aquatic stages for growth (eggs, larvae, pupae and emergence) and four aerial stages for breeding among adults (mating, blood feeding, gestating and ovipositing) [25]. To simplify the entire life-cycle, our model merges the emergence stage and the mating stage into the stage of emerging adults, while other seven sub-stages are retained, as shown in Fig. 1b. In addition, to further account for the effect of diapause, the abundance of *Ae. albopictus* eggs are divided into two groups: non-diapause eggs and diapause eggs to represent two distinct hatching behaviors.

Temperature plays an indispensable role in mediating the development and mortality rate of the *Ae. albopictus* throughout the life-cycle stages; and the aquatic period is significantly affected [38]. Erickson et al. [23] firstly developed a basic six-staged population model to describe this temperature-driven mechanism. Meanwhile, Cailly et al. [24] introduced diapause and also accounted for temperature as the major climatic trigger using a ten-staged population model. In addition, the second climatic mediator refers to precipitation, which contributes to the environmental carrying capacity (defined as the maximum population abundance the environment can sustain) at both the larval stage and the pupal stage [12, 39–41]. Based on Cailly et al. [24], Tran et al. [27] further considered both temperature and precipitation in the development of *Ae. albopictus* by a ten-stage mechanistic model. Based on these three models [23, 24, 27], an equivalent seven-stage model is proposed as the basis of the paper, as given in Equation 2. In this equation, $\dot{X}$ denotes a variable *X* taking a derivative with respect to time *t* in day of year. This equation denotes the daily variation of population abundance at each of the seven sub-stages including eggs (*E*), larvae (*L*) and pupae (*P*) in the aquatic period as well as emerging adults (*A*_em_), blood feeding adults (*A*_b_), gestating adults (*A*_g_) and ovipositing adults (*A*_o_) in the aerial period. In general, this model specifies that the population at each sub-stage is composed of (1) change of the population at the current stage (determined by the mortality rate and development rate) and (2) the population accumulated from the previous stage. In Equation 2, some parameters are dependent on climatic variables including temperature (*T*) and precipitation (*P*), as described in Table 2; other parameters independent of *T* and *P* are given by experiments from existing literature, as given in Table 3.

**Table 2:** Parameters dependent of climatic variables in the model

Parameter	Definition	Reference
*f_*E*_	Egg hatching rate (day^-1^)	Dependent of *T* [47, 50]
*f_*L*_	Larval development rate (day^-1^)	Dependent of *T* [47]
*f_*P*_	Pupal development rate (day^-1^)	Dependent of *T* [47]
*m_*L*_	Larval mortality rate (day^-1^)	Dependent of *T* [13]
*m_*P*_	Pupal mortality rate (day^-1^)	Dependent of *T* [13]
*m_*A*_	Adult mortality rate (day^-1^)	Dependent of *T* [13]
*β	Oviposition rate by each female (day^-1^)	Dependent of *T* [53]
*f*_*Ag*_	Gestating adult development rate (day^-1^)	Dependent of *T* [47, 50]
*k*_*L*_	Environmental carrying capacity for larvae (ha^-1^)	Dependent of *P*, *κ*_L_ [39–41]
*k*_*P*_	Environmental carrying capacity for pupae (ha^-1^)	Dependent of *P*, *κ*_P_ [39–41]

**Table 3:** Parameters independent of climatic variables in the model

Parameter	Definition	Value	Reference
*κ*_L_	Standard environmental carrying capacity for larvae (ha^-1^)	250,000	[24, 27]
*κ*_P_	Standard environmental carrying capacity for pupae (ha^-1^)	250,000	[27]
*σ*	Percentage of females at emergence stage	0.5	[47]
*m*_*E*_	Egg mortality rate (day^-1^)	0.05	[23, 27]
*μ*_*em*_	Emerging adult mortality rate (day^-1^)	0.1	[27]
*μ*_*r*_	Adult mortality rate related to seeking behavior (day^-1^)	0.08	[27]
*γ*_*Aem*_	Emerging adult development rate (day^-1^)	0.4	[27]
*γ*_*Ab*_	Blood feeding adult development rate (day^-1^)	0.2	[27]
*γ*_*Ao*_	Ovipositing adult development rate (day^-1^)	0.2	[27]

2 $$ \left\{\begin{array}{l}\overset{.}{E}=\beta {A}_o-\left({m}_E+{z}_{dia}{f}_E\right)E\\ {}\overset{.}{L}={z}_{dia}{f}_EE-\left[{m}_L\left(1+L/{k}_L\right)+{f}_L\right]L\\ {}\overset{.}{P}={f}_LL-\left({m}_P+{f}_P\right)P\\ {}{\overset{.}{A}}_{em}={f}_P\sigma {e}^{-{\mu}_{em}\left(1+P/{k}_P\right)}P-\left({m}_A+{\gamma}_{Aem}\right){A}_{em}\\ {}{\overset{.}{A}}_b=\left({\gamma}_{Aem}{A}_{em}+{\gamma}_{A_o}{A}_o\right)-\left({m}_A+{\mu}_r+{\gamma}_{Ab}\right){A}_b\\ {}{\overset{.}{A}}_g={\gamma}_{Ab}{A}_b-\left({m}_A+{f}_{Ag}\right){A}_g\\ {}{\overset{.}{A}}_o={f}_{Ag}{A}_g-\left({m}_A+{\mu}_r+{\gamma}_{Ao}\right){A}_o\\ {}{z}_{dia}=\left\{\begin{array}{l}0,\kern0.5em \mathrm{during}\kern0.5em \mathrm{diapause}\\ {}1,\kern0.5em \mathrm{otherwise}\end{array}\right.\end{array}\right. $$

This proposed basic model is still in need of further scrutiny, in that it overlooks two important mechanistic facets. First, as noted by Cailly et al. [24, 27] and Tran et al. [27], diapause characterized only by a single binary variable (*z*_dia_) appears to be flawed. When applied for a different region, this model is less effective by assuming a static diapause period, say late September through early March (*z*_dia_ = 0), and the rest of the year as the non-diapause period (*z*_dia_ = 1) [27]. As diapause is a climate-driven phenomenon that differs across geographic regions and arises on different days [7], a model that considers the temporal variation of diapause needs to be considered and validated for all life-cycle stages of *Ae. albopictus*. Secondly, when the effect of diapause is incorporated, the role of climate-dependent parameters (Table 2) needs to be further scrutinized toward a better model performance. To address these two concerns, we propose an improved mechanistic population model that aims to adjust asterisked parameters in Table 2.

### Improved mechanistic population model

In this section, we focus on improving the basic model (Equation 2) from two perspectives: model structure and model parameters. First, we integrate the confirmed relationship between diapause-related effects and climatic variables (temperature and photoperiod); and then we restructured the model by quantifying the conditions when the diapause arises. Secondly, we adjust several model parameters, such as development rates, mortality rates and oviposition rate (see asterisked parameters in Table 2).

#### Model structure: relationship between diapause and climate

In our field work, the absence of larvae throughout most winters indicates the likelihood of diapause in the two Chinese cities (Table 1). For the study of *Ae. albopictus,* the pressing need is to quantify the climatic thresholds under which diapause occurs and how diapause contingently influences subsequent development. Based on former experimental findings [31, 42], a female chooses to lay diapause eggs when two following conditions are simultaneously fulfilled: (1) the temperature is below 21 °C and (2) the length of daylight hours is around 13h/14h. Based on this conclusion, we denote a binary variable *z*_1_(*t*) as the state of diapause egg oviposition at day *t* of the year (Equation 3). This variable is dependent on the average temperature (*T*_aver_) and the average daylight hours (*D*_aver_) observed in the week before *t*. As the oviposition takes place normally in early autumn [7], here we consider that the oviposition process starts in the beginning of autumn (*t*_1_, August 31 or the 243th day of year [DOY]) and ends in the day when the diapause period begins (*t*_begin_).

3 $$ {z}_1(t)=\left\{\begin{array}{l}1,{T}_{\mathrm{aver}}(t)<{21}^{\circ}\mathrm{C}\kern0.5em \mathrm{and}\kern0.5em {D}_{\mathrm{aver}}(t)<13.5\mathrm{h},{t}_1<t<{t}_{begin}\\ {}0,\mathrm{otherwise}\end{array}\right. $$

For simplicity, here we denote the percentage of diapause eggs (*r*_dia_) as *E*_dia_/*E*, where *E = E*_0_ + *E*_dia_ (*E*_0_ is the number of non-diapause eggs and *E*_dia_ is the number of diapause eggs). According to Tran et al. [27], when a significant portion of diapause eggs are oviposited (*r*_dia_ > 0.9), the species steps into its diapause period at *t*_begin_. The diapause continues and ends when these eggs begin to hatch. As observed in Toma et al. [43], the diapause eggs did not hatch until weekly mean temperature rose to 10 °C/11 °C and the daylight hours reached 11h/11.5h. Based on the evidence, we denote another binary *z*_2_(*t*) to control for the climatic threshold for the hatching event, indicating the end of egg diapause at day *t* of year (Equation 4), where *t*_end_ is the day when the diapause period ends and *t*_2_ is the first day of year when *r*_dia_ (t) < 0.1.

4 $$ {z}_2(t)=\left\{\begin{array}{l}1,{T}_{\mathrm{aver}}(t)>{10.5}^{\circ}\mathrm{C}\kern0.5em \mathrm{and}\kern0.5em {D}_{\mathrm{aver}}(t)>10.25\mathrm{h},\kern0.5em {t}_{end}<t<{t}_2\\ {}0,\kern0.5em \mathrm{otherwise}\end{array}\right. $$

During the diapause period (*t*_begin_ < *t* < *t*_end_), on average *Ae. albopictus* adults succumb to a temperature of under 9.5 °C (*γ*_*Aem*_ *= γ*_*Ag*_ *= γ*_*Ar*_ *=* 0 when *T*_aver_ < 9.5 °C) [43].To survive such extreme conditions, egg diapause occurs as an adaptive strategy to improve survival rate compared with non-diapause egg [7, 34, 35]. However, the mortality rate and development rate of diapause eggs are less explored by existing literature; and therefore we denote these two parameters as *m*_dia_ and *f*_dia_ respectively in Equation 5 and conduct their sensitivity analysis in Results section. In addition to Equation 4, the hatching of diapause eggs is subject to other environmental factors, such as water and food availability [32, 44, 45]. In accordance with Tran et al. [27], we consider that the hatching is also dependent on the first precipitation event in spring (*P*_week_ > *P*_0_, where *P*_0_ = 0 mm).

With these improvements applied in Equation 2, the adjusted model is presented in Equation 5 to characterize the population dynamics of *Ae. albopictus* in a seven-stage development process, where the notations follow Tables 2 and 3. This model features the temperature- and photoperiod- driven mechanism in both the oviposition *z*_1_(*t*) and the egg diapause *z*_2_(*t*), while considering diapause egg survival and hatching during the diapause period.

5 $$ \begin{array}{l}\left\{\begin{array}{l}{\overset{.}{E}}_0=\left(1-{z}_1\right)\beta {A}_o-\left({m}_E+{f}_E\right){E}_0\\ {}{\overset{.}{E}}_{dia}={z}_1\beta {A}_o-\left({m}_{dia}+{z}_2\;{f}_{dia}\right){E}_{dia}\\ {}\overset{.}{L}=\left({f}_E{E}_0+{z}_2\;{f}_{dia}{E}_{dia}\right)-\left[{m}_L\left(1+L/{k}_L\right)+{f}_L\right]\kern0.1em L\\ {}\overset{.}{P}={f}_LL-\left({m}_P+{f}_P\right)P\\ {}{\overset{.}{A}}_{em}={f}_P\sigma {e}^{-{\mu}_{em}\left(1+P/{k}_P\right)}P-\left({m}_A+{z}_{dia}{\gamma}_{Aem}\right){A}_{em}\\ {}{\overset{.}{A}}_b={z}_{dia}\left({\gamma}_{Aem}{A}_{em}+{\gamma}_{Ao}{A}_o\right)-\left({m}_A+{\mu}_r+{z}_{dia}{\gamma}_{Ab}\right){A}_b\\ {}{\overset{.}{A}}_r={z}_{dia}{\gamma}_{Ab}{A}_b-\left({m}_A+{f}_{Ag}\right){A}_g\\ {}{\overset{.}{A}}_o={f}_{Ag}{A}_g-\left({m}_A+{\mu}_r+{z}_{dia}{\gamma}_{Ao}\right){A}_o\\ {}E={E}_0+{E}_{dia}\end{array}\right.\\ {}\mathrm{where}\kern0.5em {z}_{dia}(t)=\left\{\begin{array}{l}0,{T}_{ave}(t)<{9.5}^{\circ}\mathrm{C},\kern0.5em {t}_{begin}\le t\le {t}_{end}\\ {}1,\kern0.5em \mathrm{otherwise}\end{array}\right.\\ {}{t}_{begin}=\left\{\left.t\;\right|\kern0.24em {r}_{dia}(t)>0.9\kern0.62em \mathrm{in}\kern0.5em \mathrm{the}\kern0.5em \mathrm{third}\kern0.5em \mathrm{quarter}\kern0.5em \mathrm{of}\kern0.5em \mathrm{the}\kern0.5em \mathrm{year}\right\}\\ {}{t}_{end}=\left\{\left.t\;\right|\kern0.24em {z}_2(t)=1\kern0.5em \mathrm{in}\kern0.5em \mathrm{the}\kern0.5em \mathrm{first}\kern0.5em \mathrm{quarter}\kern0.5em \mathrm{of}\kern0.5em \mathrm{the}\kern0.5em \mathrm{year}\right\}\kern2em .\end{array} $$

#### Model parameters: temperature-driven mechanism

To derive the asterisked parameters in Table 2, we have conducted a separate set of controlled experiments to investigate the response of *Ae. albopictus* to different temperatures in the aquatic period [46]. In these experiments, the development lengths of eggs, larvae and pupae, and the survival rate of larvae under different temperatures (16 °C, 21 °C, 26 °C, 31 °C and 36 °C) have been identified (Table 4). Unfortunately, due to limited laboratory conditions, several other records, such as the survival rate of pupae, could not be obtained. Therefore, we have derived these missing variables from existing literature as a compromise solution [7, 47, 48], as shown in Table 4.

**Table 4:** Development lengths and mortality rates of *Ae. albopictus* under different temperatures

Temperature (°C)	5	10	11	15	16	17	20	21	24	25	26	27	30	31	35	36
Egg hatching length (days)	11.0^b^	2.0^b^	8.8^d^	7.4^b^	3.4^d^	6.0^a^	6.0^a^	2.5^d^	3.0^a^	3.0^a^	1.9^d^	2.0^a^	2.0^a^	2.0^d^		2.4^d^
Larval development length (days)				26.9^b^	21.6^d^		11.0^a^	9.9^d^		7.0^a^	6.3^d^	6.1^a^	5.7^a^	5.5^d^	12.1^b^	12.0^d^
Pupal development length (days)				8.7^b^	4.3^d^		4.1^b^			2.7^a,b^	2.2^d^	2.1^a^	1.9^b^	1.6^d^	1.7^b^	
Larval survival rate (%)^b^				59.5			86.3			81.2			75.1			30.0
Pupal survival rate (%)^b^				83.3			89.9			93.8			90.0			
Adult survival length (days) ^e,c^	9	23		36			42			44			40		23	

Note: A blank slot indicates no observed data under this temperature

^a^Data derived from Hawley [7];

^b^Data derived from Delatte *et al*. [47];

^c^Data derived from Oliver *et al*. [48];

^d^Data derived from Yu [46];

^e^Adult survival length is defined as the period during which survival rate is over 50 %

**Fig. 2:**
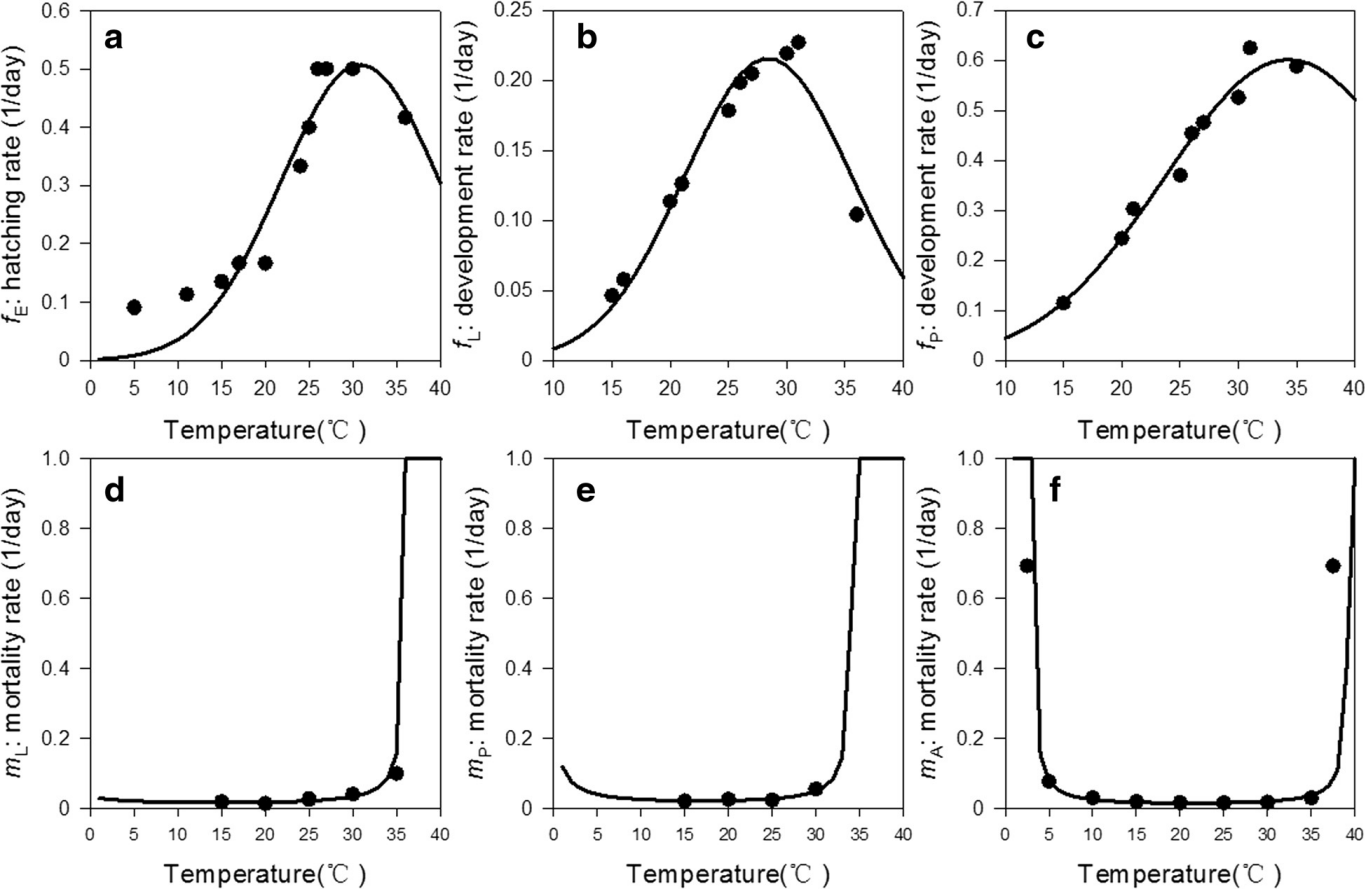
Temperature-dependent parameters at different development stages: **a** egg hatching rate, **b** larval development rate, **c** pupal development rate, **d** larval mortality rate,** e** pupal mortality rate, and **f** adult mortality rate

We then describe these data in a scatter diagrams: the hatching rate (*f*_E_), the larval development rate (*f*_L_) and the pupal development rate (*f*_P_) as the reciprocal of the development length under different temperatures (*T*), respectively (Figs. 2a-c). Previous studies have explored a linear or a quadratic equation [27] to describe the relationship between the development rates (*f*_X,_*X = E*, *L*, *P*) and temperature *T*. Our preliminary analysis has identified a better fit with the Gaussian function. For each stage *X*, *f*_X_ is formulated by the Gaussian curve as a dependent of *T*, as shown in Equation 6. Coefficients and fitting *r*-squares for each stage *X* are given in Table 5. This relationship describes a biological pattern that *f*_X_ gradually increases at a low temperature, reaches a maximum in an optimal temperature range (28–34 °C), and then drops to zero at a lethal high temperature.

**Table 5:** Coefficients and *r*-square of the fitting equations (Equations 6 and 7)

Parameter	Coefficient a	Coefficient b	Coefficient c	*r*-square
*f*_E_(*t*)	0.5070	30.85	12.82	0.9081
*f*_L_(*t*)	0.1727	28.40	10.20	0.9616
*f*_P_(*t*)	0.6020	34.29	15.07	0.9730
*m*_L_(*t*)	-0.1305	3.868	30.83	0.8252
*m*_P_(*t*)	-0.1502	5.057	3.517	0.8340
*m*_A_(*t*)	-0.1921	8.147	-22.98	0.9317

6 $$ {f}_x={f}_x(T)=aexp\left[-{\left(\frac{T-b}{c}\right)}^2\right]. $$

To explore the relationship between the larva/pupa mortality rate (*m*_X_, *X* = *L, P*) and temperature, we explored functions established in other literature through trial and error, such as the monotonous exponential function (*m*_X_ = *e*^-*T*/2^_+_ constant) [27] and the linear function (*m*_X_ = *aT*_+_ constant, *X* = *A*) [13]. However, none of these functions fit well with our observations. Our collected data showed that the mosquito had relatively higher mortality rates under high and low temperatures (Fig. 2d-f) [47, 48]. Based on this finding, a new regression function between the *m*_X_ and *T* is established, where *m*_X_ is adjusted to the range of [0, 1], as shown in Equation 7 and Table 5. This equation provides a better fit to our observed population abundance and is relatively consistent with the regression pattern of the mosquito’s survival rate [49–52]. Appendix A describes the process of establishing this equation in greater detail.

7 $$ {m}_x={m}_x(T)= \min \left\{\frac{1}{\left|a{T}^2+bT+c\right|},1\right\}. $$

In addition to the temperature-driven mechanism of development rates and mortality rates, we have adopted an established relationship between the egg ovipostion rate and temperature proposed by Yang et al. [53], as given in Equation 8. This equation is employed to substitute the oviposition rate in Tran et al.’s model [27], whereas in their model the rate was formulated as a constant.

8 $$ \beta =\beta (T)= \max\ \left\{-15.837{T}^2+1.2897T-0.0163,0\right\}. $$

## Results

The scientific significance of the proposed model can only be corroborated by rigorous validation with respect to field data. To prepare for validation, we aimed to derive the simulation results first with an initial population of 10^6^ eggs and a starting time *t*_0_ of January 1^st^. Then the model was discretized using the Euler Method in MatLab 2015 [54]. Specifically, multiple rounds of simulations were conducted on a daily basis over six years in Guangzhou (2006–2011) and Shanghai (2008–2013). The results derived for the first year (i.e. 2006 for Guangzhou and 2008 for Shanghai) were not used for comparison, as they were strongly dependent on the initial setting [24, 27].

We then compared larva abundance between the monthly observed CI (Table 1) and the monthly simulated results (*L*_R_, which is relative to the maximum simulated value over the entire study period) using the Pearson’s correlation coefficient (*r*). In this case, the study period is 2007–2011 for Guangzhou and 2009–2013 for Shanghai.

### Simulated diapause periods

**Fig. 3:**
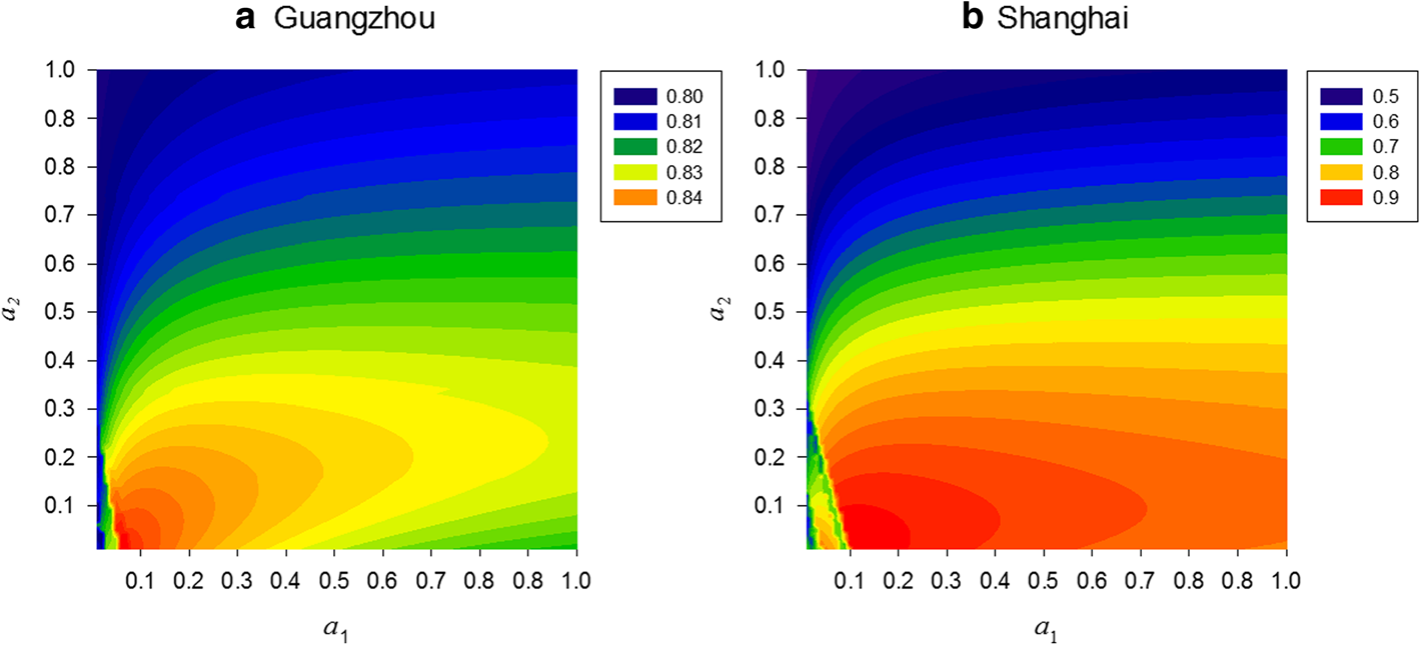
Correlation coefficient (*r*) with different combinations of *a*_1_ (0.01–1.00) and *a*_2_ (0.01–1.00) for **a** Guangzhou and **b** Shanghai

In the model, two parameters *m*_dia_ and *f*_dia_ related to diapause egg survival remained undetermined. To derive this set of parameters, we compared them with *m*_E_ and *f*_E_ in the original model (Equation 2): *m*_dia_ = *a*_1_*m*_E_ and *f*_dia_ = *a*_2_*f*_E_ where 0 < *a*_1_ < 1, 0 < *a*_2_ < 1; and then we traversed *a*_1_ and *a*_2_ in a given range (*a*_1_: 0.01–1, *a*_2_: 0.01–1) and derived the optimal combination with the highest *r* for each city, as shown in Fig. 3. The highest *r* was chosen as it represented the best fit between the field data and the simulation results, eventually yielding the best model performance. In Guangzhou, the *r* is comparatively stable within a narrow range of 0.80–0.85 (Fig. 3a), whereas in Shanghai, *r* fluctuates over a wider range of 0.5–0.9 (Fig. 3b). The highest *r* was derived at *a*_1_ = 0.1 and *a*_2_ = 0.1 for both cities (at *r* = 0.84 for Guangzhou and *r* = 0.90 for Shanghai). Based on the optimal combination, we estimated the starting day and ending day of the diapause with the new model.

**Fig. 4:**
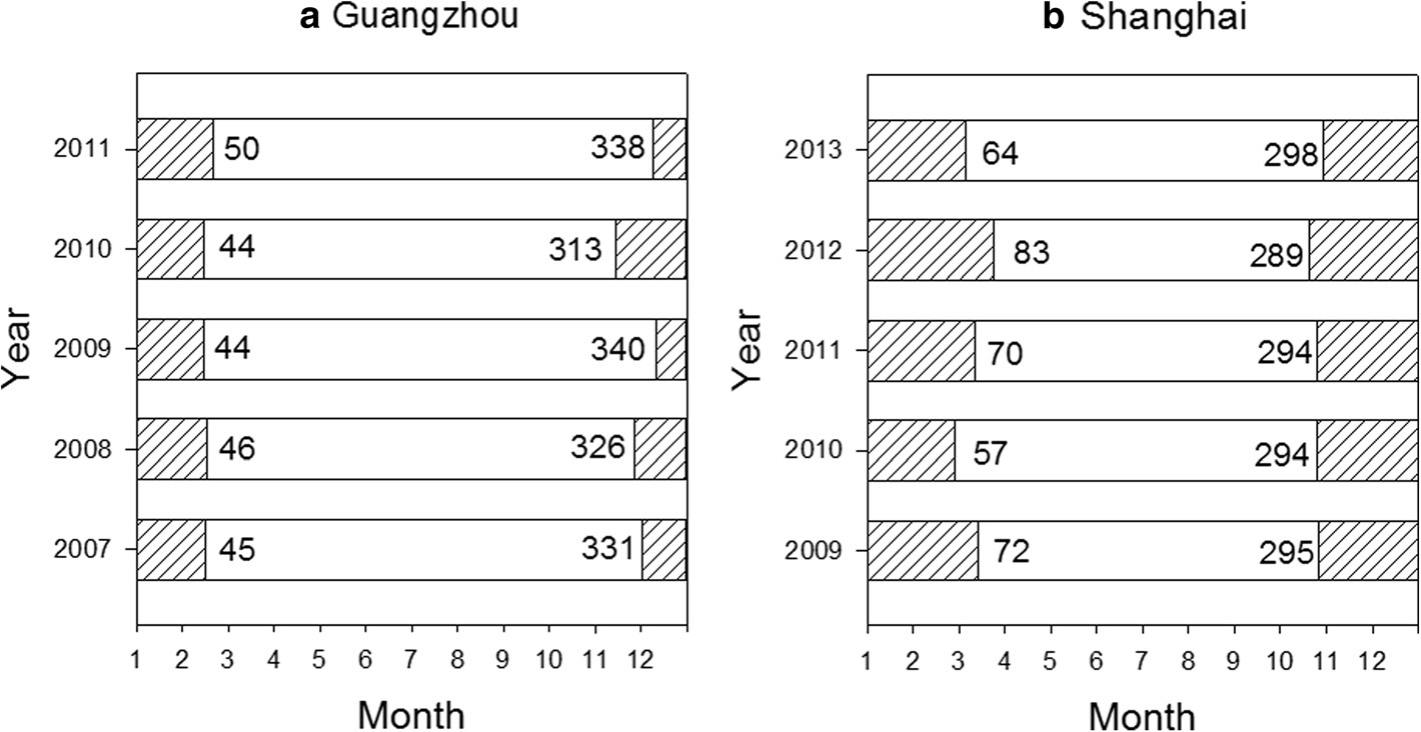
The simulated diapause period (shaded areas) and non-diapause period (white areas) of *Ae. albopictus* population for **a** Guangzhou, 2007–2011 and **b** Shanghai, 2009–2013. The numbers represent the starting DOY (*t*_*b*_) and the ending DOY (*t*_*e*_) of the diapause period

Figure 4 summarizes the finding about the diapause period and the non-diapause period with starting time (*t*_b_) and ending time (*t*_e_) in both cities. It shows that on average, the diapause in Guangzhou began in late November on the 330st day of the year (DOY 330) and ended in early March the next year (DOY 46); and in Shanghai, it began in mid-October (DOY 294) and ended in mid-March the next year (DOY 69). This result indicates an average diapause period of 81 days in Guangzhou and 140 days in Shanghai.

### Simulated population abundance

**Fig. 5:**
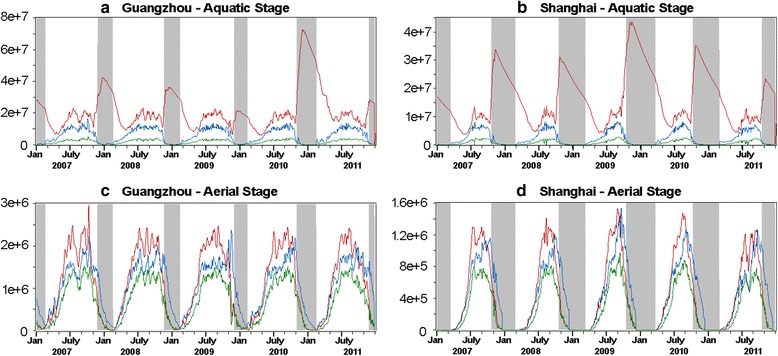
Simulated *Ae. albopictus* population over a respective five-year period for Guangzhou (2007–2011) and Shanghai (2009–2013) with respect to: **a**-**b** eggs (*red*), larvae (*blue*) and pupae (*green*); **c**-**d** blood feeding adults (*red*), gestating adults (*blue*) and ovipositing adults (*green*). Grey areas are simulated diapause periods

Based on the highest *r* derived at *a*_1_ = 0.1 and *a*_2_ = 0.1, we simulated the population abundance for each of the seven stages on a daily basis. Figure 5 shows part of the results in the aquatic period (*E, L, P*) and the aerial period (*A*_b_, *A*_g_, *A*_o_). A general observation is that the simulated population at each stage is nearly two times greater in Guangzhou (2007–2011) than that in Shanghai (2009–2013). This result might be attributed to the fact that the duration of the non-diapause period is longer in Guangzhou, which creates a more favorable environment for the mosquito to survive and develop. We then estimated the favorable development period at the larval-pupal stage (*L* + *P*) and at the adult stage (*A*_b_ + *A*_o_) for the two cities on an annual basis, as given by Table 6.

**Table 6:** Simulated favorable development period (DOY: *F*_LP_ or *F*_A_ >1%) at the larval-pupal stage (*L* + *P*) and at the adult stage (*A*_b_ + *A*_o_)

Guangzhou	2007	2008	2009	2010	2011	Average
Larval-pupal (*F*_LP_ > 1%)	46–352	47–353	45–365	45–335	47–354	46–352 (Feb–Dec)
Adult (*F*_A_ > 1%)	58–358	73–358	60–364	60–341	65–357	63–356 (Mar–Dec)
Shanghai	2009	2010	2011	2012	2013	Average
Larval-pupal (*F*_LP_ > 1%)	77–341	62–324	73–313	87–315	75–330	75–325 (Mar–Nov)
Adult (*F*_A_ > 1%)	114–320	127–320	119–315	121–309	120–318	120–356 (Apr–Nov)

*F*_LP_: percentage of larval-pupal population in total population across all stages of a year;

*F*_A_: percentage of blood feeding and ovipositing adults in total population across all stages of a year

### Model validation

**Fig. 6:**
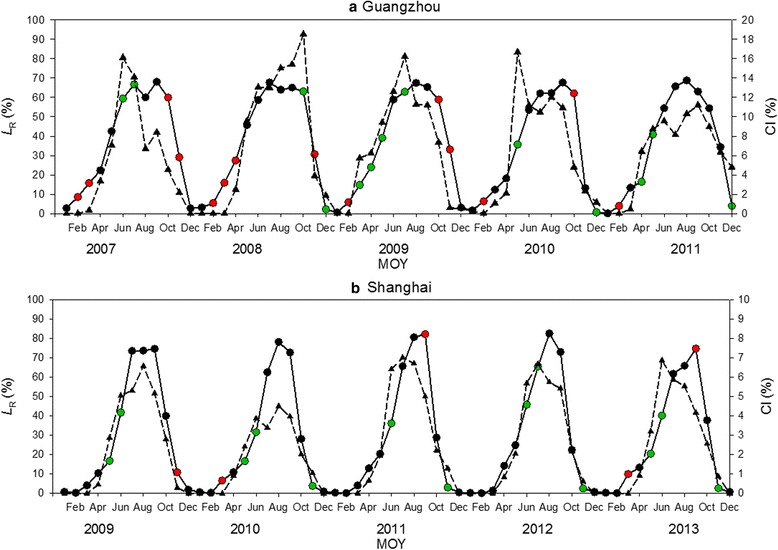
Comparison between observed monthly Container Index (CI, dotted lines) and simulated monthly relative abundance of larvae (*L*_R_, solid black lines) for **a** Guangzhou, 2007–2011 and **b** Shanghai, 2009–2013. Green dots represent underestimation of the model and red dots represent overestimation

**Fig. 7:**
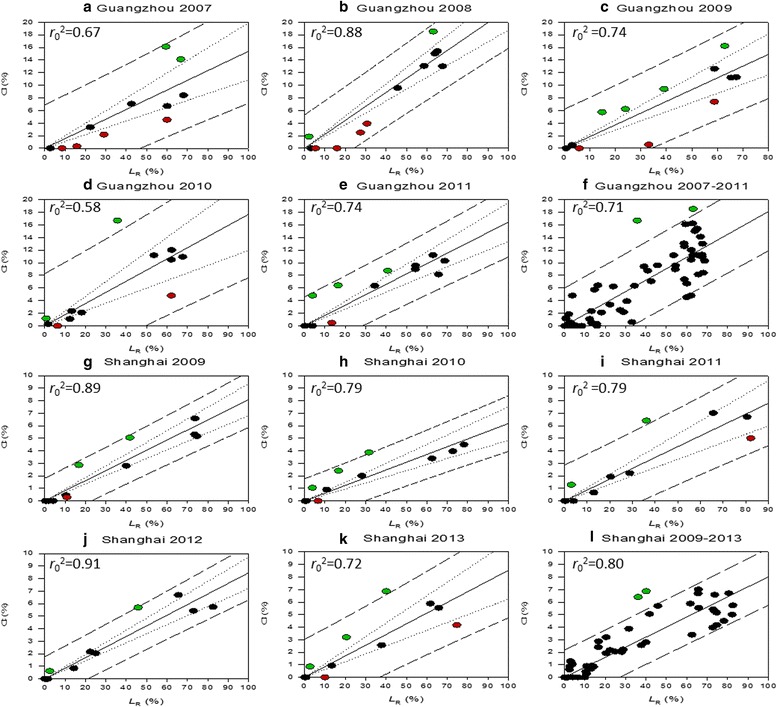
Zero-intercept *r*-square values (*r*_0_^2^) derived from the regression analysis between observed monthly larval Container Index (CI) and simulated monthly relative larval abundance (*L*_R_) for **a**-**f** Guangzhou, 2007–2011 and **g**-**l** Shanghai, 2009–2013. Green dots represent underestimation of the model and red dots represent overestimation. Dashed lines (-▪-) are thresholds of the predicted interval, while dotted lines (▪▪▪) are thresholds of the confident interval

The validation of the model is shown in Figs. 6 and 7, using the correlation coefficient (*r*) and zero-intercept *r*-square (*r*_*0*_^*2*^), respectively.

Figure 6 is the comparison between the observed CI and the simulated *L*_R_ over a five-year period in Guangzhou and that in Shanghai (Fig. 6). The simulated population is highly consistent with the field observations (CI), with *r* equals to 0.84 for Guangzhou (Fig. 6a) and 0.90 for Shanghai (Fig. 6b). However, a good match with field data does not fully capture the fluctuation of population, leading to underestimations (green dots) and overestimations (red dots) for certain months. Four apparent underestimations are Oct 2008, May 2010 for Guangzhou and Jun 2011, Jun 2013 for Shanghai.

Figure 7 shows the *r*^2^ for the comparison within each single year. Generally, over a five-year period, Shanghai (Fig. 7l) has a better fit than Guangzhou (Fig. 7f) in terms of *r*_0_^2^. Specifically, in Shanghai, the simulation result is overall very satisfactory with the best fit appearing in 2012 (*r*_0_^2^ = 0.91, Fig. 7j); in Guangzhou, the simulation result in 2007 is considerably underestimated (*r*_0_^2^ = 0.67 compared to the average *r*_0_^2^ = 0.71 in Fig. 7f).

These results share similar findings with other case studies in Shanghai [55, 56] and Guangzhou [57, 58]. Specifically, the predicted population growth periods in Shanghai (i.e. *F*_LP_ > 1 %, March-November; *F*_A_ > 1 %, April-November, Table 6) parallel with existing studies [55, 56].

### Sensitivity analysis

**Fig. 8:**
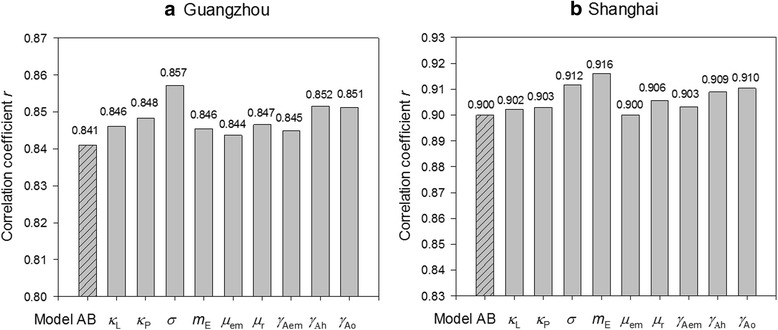
Comparison of correlation coefficients (*r*) of Model O, Model A, Model B and Model AB for **a** Guangzhou and **b** Shanghai

In addition to model validation, another pressing need is to examine the sensitivity of the new model to the change of parameters. The nine parameters independent of climatic variables (given in Table 3) were examined by using the fraction factorial design [59]. The initial values of these parameters were assigned according to existing literature [23, 24, 27, 47]. Then we reassigned a value to each of the parameters (e.g. *m*_E_) within a ±10 % range (e.g. 0.9*m*_E_-1.1*m*_E_) and derived the maximum *r* in the new model (Equation 5). We followed this method by changing each of the nine parameters, one at a time, and derived nine maximum *r*. Fig. 8 shows the results.

It can be seen from Fig. 8 that the change of *r* introduced by the single-factor sensitivity analysis is generally minor. The overall improvement of *r* ranges from 0.004 to 0.017 (or 0.4–2.0 %) in Guangzhou and from 0 to 0.016 (0–1.7 %) in Shanghai. The greatest change is observed on parameter σ in the case of Guangzhou and *m*_E_ in Shanghai. This analysis provides compelling evidence that the proposed model is relatively robust and is not sensitive to non-climatic variables, corroborating the applicability of the model.

## Discussion

This section evaluates the improvement of the new model based on several adjustments to the original model and discusses the difference in model performance between the two study areas.

### Model comparison

First, we would like to examine the improvement of the new model (designated as Model AB) by comparing with the original model (Model O) and two partially adjusted models (Model A and Model B) according to the Methods section.

- *Model O*: Original model (Equation 2)
- *Model A*: Original model with diapause-related structural adjustment (Equation 5)
- *Model B*: Original model with temperature-related parameter adjustment (Equation 2 + Equations 6–8)
- *Model AB*: New model with all adjustments applied (Equation 5 + Equations 6–8)

**Fig. 9:**
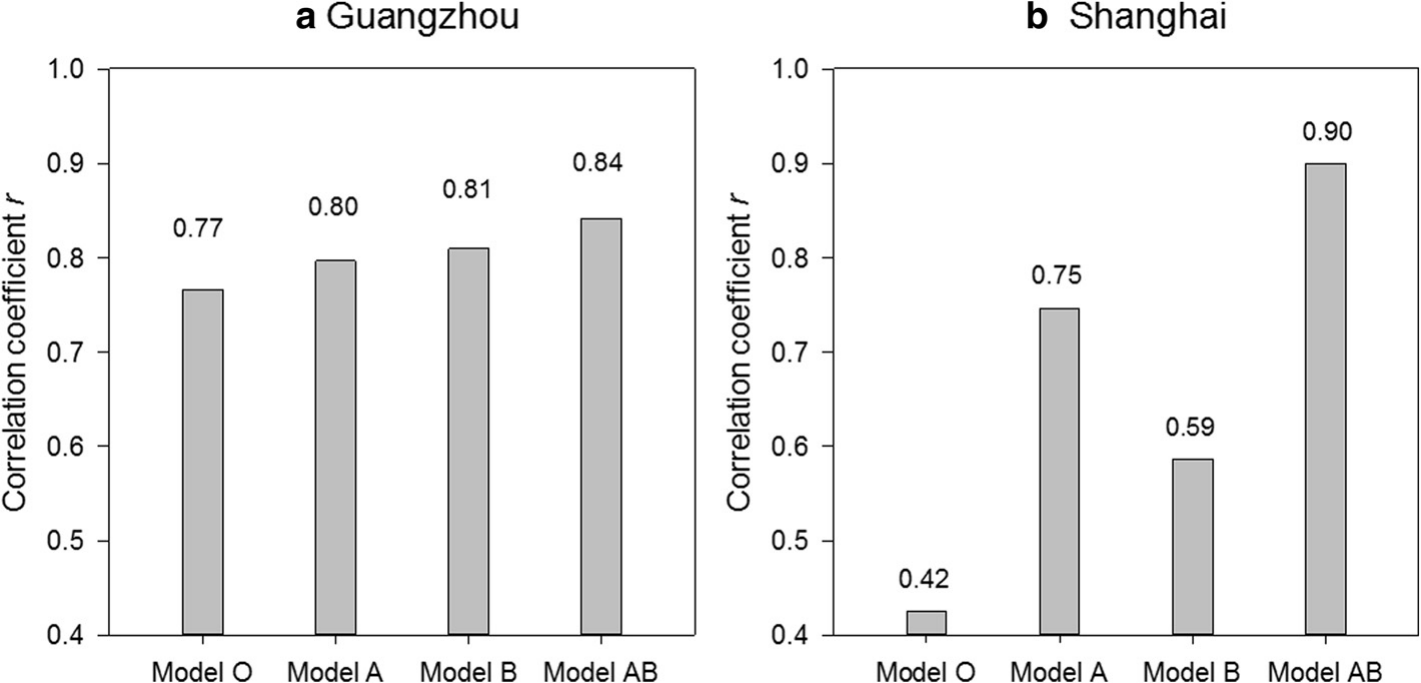
Correlation coefficients *r* generated by single-factor adjustment in Model AB for **a** Guangzhou and **b** Shanghai. The x-axis includes nine non-climatic parameters, as given in Table 3. The grey bar is the maximum fitting *r* by adjusting that parameter within a ±10 % range; and the shaded bar is the *r* of the new model without any adjustment

Figure 9 shows the correlation coefficient *r* with the two sets of field data applied into these four models. It can be seen from the comparison that although Model O is well suited for Guangzhou (*r* = 0.77), it does not perform well for Shanghai (*r* = 0.42). This result is possibly introduced by the static diapause parameters in Model O that could not fully capture the climatic-driven mechanism of diapause, degrading the performance of the model. When the diapause effect is included, Model A demonstrates a huge improvement in cases of Shanghai (*r* = 0.75 or an improvement of 78.6 %), where diapause shows a confirmed seasonal pattern. Adjusting the model with only temperature-related parameters (Model B) yields moderate improvement in both study areas; and the best performance is achieved with two facets of adjustments applied.

### Regional comparison

The results above corroborate the model’s potential in simulating *Ae. albopictus* population. However, compared to Shanghai (31.2°N), the performance in Guangzhou (23.1°N) seems to be less satisfactory (i.e. curve fitting rate is 90 % for Shanghai and 84 % for Guangzhou, Fig. 6). This regional difference could be explained in two very different respects.

First, the effect of the diapause in the low-latitude area is of arguable existence [7]. Guangzhou is located further south and has comparatively warm and humid winters. The mild climatic conditions could be favorable for certain *Ae. albopictus* to sustain and develop without taking the strategy of diapause. For example, our field experiments showed evidence that during winter times there were still a very low number of larvae, pupae and adults (Fig. 5) in Guangzhou that could circumvent the process of diapause. This observation was supported by Liu et al. [58] that found in the same region a small proportion of *Ae. albopictus* larvae hatched after mid-November could still survive the extreme weather and grow into non-ovipositing adults. A separate evidence could be found in Fig. 9 that applying the diapause-related structural adjustment is less effective in Guangzhou (r: 0.72 → 0.80) than in Shanghai (r: 0.42 → 0.75).

Secondly, the seasonality of *Ae. albopictus* is relatively complex in tropical climate areas. In an annual development cycle, *Ae. albopictus* population showed one peak in subtropical climate areas and two peaks with different magnitudes in tropical climate areas [60]. As Guangzhou is located in close vicinity to tropical climate areas, our field observations of CI show interchangeable patterns (i.e. 2007, 2010 and 2011 have two peaks, while 2008 and 2009 have one peak, Fig. 6a). This dynamic pattern created a certain degree of uncertainty for validation and eventually degraded model performance for cases in Guangzhou. Comparatively, Shanghai with an apparent single-peak pattern in the years of study yielded better simulation results (Fig. 6b).

## Conclusions

The phenomenon of diapause among *Ae. albopictus* has been confirmed in most temperate climate areas and is of arguable existence in subtropical climate areas [7]. Generally, low temperatures and short photoperiods create an unfavorable condition for *Ae. albopcitus* to develop. To survive the extreme conditions, especially during winter times, egg diapause occurs as an adaptive strategy to lower mortality rates [1, 7]. This climate-driven mechanism of diapause has only been explored in a limited manner [24, 27].

This paper proposes a climate-driven mechanistic population model of *Ae. albopictus* that accounts for the biological phenomenon of diapause. The model is a natural extension of two existing mechanistic population models [24, 27] with emphasis on the climate-driven diapause conditions and stage-specific moderating variables. Although the former models also considered diapause, several issues remained unaddressed, such as identifying the time when diapause eggs are laid and hatched, demarcating the thresholds of diapause and non-diapause periods, and incorporating the mortality rate and the hatching rate of diapause eggs. To remedy these flaws, an improved generic model is proposed, capturing the multifaceted climate-driven mechanism of diapause-related effects. The formation of the model and the attribution of parameters are fine-tuned to existing research and field data collected in two Chinese cities over a respective five-year period. Overall, the simulation results are relatively compelling and fit the majority of our field observations. The study also confirms the respective as well as the joint effects of model structure and temperature-driven parameters, corroborating findings from other mechanistic models [23, 24, 27].

Admittedly, the proposed model is methodologically flawed in several aspects. First, the parameter of precipitation was included but was not closely examined in our model. Existing studies have identified either a positive [41], a negative [12], or no influence of precipitation on population abundance [43]. The actual effect of precipitation should be carefully weighted through rigorous field observations before any attempt to quantify the variable in the model. Secondly, in the study only the Container Index was used for model validation and could not fully represent the multifaceted population growth of *Ae. albopictus*. Future research should explore other monitoring indices, such as the Breteuil Index (BI) and the Housing Index (HI) to generate a solid and robust conclusion. Thirdly, the sensitivity analysis on the non-climatic variables (Table 3) is exclusive of other variables, while the joint effect of multiple variables is not evaluated. In the future the extension of the assessment should include multivariate analysis methods, such as the Fourier amplitude sensitivity testing [59, 61].

Lastly, a promising direction to extend the study considers the incorporation with other non-mechanistic ecological models, such as the GARP [62] model and the CLIMEX model [63] and the correlation with epidemic models, such as the SIR model [64, 65] to describe the mechanistic transmission and the spatiotemporal distribution of mosquito-borne epidemics. For example, one such an attempt is the work conducted by Erickson et al. [66] that integrated the research on *Ae. albopcitus* population [23] with an SEIR model [66]. To this end, better understanding the mechanism and variables effecting the *Ae. albopictus* population growth and improving the model performance will eventually contribute to proactive strategies to predict and prevent contingent mosquito-borne epidemics.

## Appendix. Formulation of the mortality rate at the larval stage and the pupal stage

To establish Equation 7, we first estimated the daily survival rate at the larval stage and at the pupal stage with the existing dataset (Table 4). Specifically, *S*_X_(*t*) denotes the survival rate at time *t* at stage *X* (larval or pupal) and *S*_X_(*t* → *t* + *d*) denotes the total survival rate from day *t* to day (*t* + *d*). Supposing *S*_X_(*t*) was stable from day (*t*) to day (*t* + *d*), *S*_X_(*t*) was calculated by Equation 9. Within the time length of *S*_X_(*t → t + d*), the daily survival rate at stage *X* under different temperatures was calculated (Table 5). According to Lunde *et al.* [67], we also obtained the daily mortality rate *m*_X_(*t*) at stage *X* under the corresponding temperature by Equation 10.

9 $$ {S}_X(t)=\sqrt[d]{S_X\left(t\to t+d\right).} $$

10 $$ {m}_X(t)=\Big\{\begin{array}{l}-1\mathrm{n}\left({S}_X(t)\right),{S}_X(t)>0,\\ {}\kern2em 1\kern2.12em ,{S}_X(t)=0.\end{array}\operatorname{} $$
